# Development of a novel clinimetric tool: PAtient Reported Disease Activity Index in Rheumatoid Arthritis (PARDAI-RA) by PANLAR, for the assessment of patients living with rheumatoid arthritis

**DOI:** 10.1007/s10067-024-06868-w

**Published:** 2024-02-14

**Authors:** Daniel G. Fernández-Ávila, Daniela Patiño-Hernández, Socorro Moreno-Luna, Lorena Brance, Álvaro Arbeláez, Antonio Cachafeiro Vilar, Carlos Lozada, Carlos Ríos, Carlos Toro, Claudia Ramírez, Guillermo Pons-Estel, Manuel Ugarte-Gil, María Narváez, Miguel Albanese, Orlando Roa, Oscar Ruiz, Paula Burgos, Ricardo Xavier, Yurilis Fuentes, Enrique Soriano

**Affiliations:** 1 Rheumatology Division, Pontificia Universidad Javeriana – Hospital Universitario San Ignacio, Bogotá, Colombia; 2 Internal Medicina Department, Hospital Universitario San Ignacio, Bogotá, Colombia; 3 Epidemiology Department, Pontificia Universidad Javeriana, Bogotá, Colombia; 4 Rheumatology Division, Universidad Nacional de Rosario, Santa Fe, Argentina; 5 Clínica Imbanaco, Cali, Colombia; 6 Pacífica Salud, Panamá, Panama; 7 Rheumatology Division, University of Miami, Coral Gables, USA; 8 Universidad de Especialidades Espíritu Santo, Guayaquil, Ecuador; 9 Centro de Referencia en Osteoporosis y Reumatología, Cali, Colombia; 10 Rheumatology Division Keralty, Bogotá, Colombia; 11 Centro Regional de Enfermedades Autoinmunes y Reumáticas (GO-CREAR), Rosario, Argentina; 12 Universidad Científica del Sur, Lima, Peru; 13 Centro de Asistencia del CASMU, Montevideo, Uruguay; 14 Clinic Immunology and Rheumatology Department, Pontificia Universidad Católica de Chile, Santiago de Chile, Chile; 15 Rheumatology Service Hospital de Clinicas de Porto Alegre, Porto Alegre, Brazil; 16 Universidad de Oriente, Cumaná, Venezuela; 17 Rheumatology Division, Hospital Italiano de Buenos Aires, Buenos Aires, Argentina

**Keywords:** Assessment, Clinimetrics, Management, Patient-reported outcome measures, Rheumatoid arthritis

## Abstract

**Background:**

Clinical experience has shown that a single measure is not sufficient to assess disease activity in rheumatoid arthritis (RA). Various clinimetric tools are necessary to address the many clinical situations that can arise.

**Methods:**

In order to develop a comprehensive measurement tool, the Pan American League of Associations for Rheumatology searched for the most frequent measures of disease activity applied in RA by means of a semi-systematic review of the available literature.

**Results:**

We found that the most frequently reported measures of disease activity were the 28-joint Disease Activity Score, C-reactive protein, and the erythrocyte sedimentation rate, followed by patient-reported measures of pain and stiffness and many other composite indices and patient-reported outcome measures. The most frequent physician-reported sign of disease was the swollen joint count, and the most frequently self-reported feature was the increase in disease activity or flares.

**Conclusion:**

In this article, we present a new clinimetric tool developed based on expert consensus and on data retrieved from our search. Disease activity can be better assessed by combining various data sources, such as clinical, laboratory, and self-reported outcomes. These variables were included in our novel clinimetric tool.

**Table:** 

**Key Points** • *The goal of treatment of RA is to achieve the best possible control of inflammation, or even remission; therefore, disease management should include systematic and regular evaluation of inflammation and health status.* • *Clinimetric tools evaluate a series of variables (e.g., symptoms, functional capacity, disease severity, quality of life, disease progression) and can reveal substantial prognostic and therapeutic differences between patients.* • *Our clinimetric tool, which is based on a combination of data (e.g., clinical variables, laboratory results, PROMs), can play a relevant role in patient assessment and care.*

**Supplementary Information:**

The online version contains supplementary material available at 10.1007/s10067-024-06868-w.

## Introduction

Rheumatoid arthritis (RA) is a chronic inflammatory autoimmune disease that affects between 0.5 and 1% of the general population, with a higher prevalence in industrialized countries [1, 2]. The prevalence of RA has increased over the past three decades, although the severity of the disease has been declining steadily, probably because of changes in treatment paradigms and better management. However, RA still carries a significant burden for patients, public health, and society [1–5].

Clinimetrics was first introduced and defined in the 1980s as a “discipline aimed at creating indices, rating scales and other expressions to describe or measure symptoms, physical signs and other clinical phenomena” [6]. Clinimetrics also includes the psychosocial impact of disease and treatment on the individual, their family, and interpersonal relationships and on daily activities and well-being [6, 7].

The expansion of the assessment process to include the psychological, emotional, and social impact of RA has led to more widespread use of biopsychosocial perspectives and patient-reported data in our approach to the disease [8–11]. Clinimetric tools evaluate symptoms, functional capacity, disease severity, timing of clinical changes, impact of comorbidities, quality of life, and progression of illness and can reveal substantial prognostic and therapeutic differences between patients [9–12].

Disease activity measures such as the 28-joint Disease Activity Score (DAS28), the Simplified Disease Activity Index (SDAI), and the Clinical Disease Activity Index (CDAI) cover the most relevant aspects of RA in a single score [12–15]. DAS28 is used in clinical trials and routine monitoring to evaluate response to treatment and inform decisions on the need to begin or adjust treatment [12–19].

Several outcome measures have proven to be helpful in daily practice, clinical trials, and clinical epidemiology [12–28]. Many variables used to assess the activity of RA are based on disease activity measures and patient-reported outcome measures (PROMs) [17–28].

In RA, the challenge to precisely evaluate the activity of disease includes having to perform a complete physical examination, which can be difficult, particularly in situations where telemedicine is required. Previous studies have found this method of patient care to be non-inferior to regular consults [29]. But since most of the available clinimetric tools require a physician’s examination, PROMs become more relevant in order to promptly adjust treatment aiming towards the remission of the activity of disease [18–20, 30].

We describe the development of a novel comprehensive clinimetric tool to record relevant symptoms of RA and their impact on patients. The tool has the potential to be used in both daily practice and clinical trials. Therefore, at the Pan American League of Associations for Rheumatology (PANLAR), we performed a semi-systematic review for current information on clinimetrics and their use in RA by using a search strategy as well as by including articles deemed relevant to clinical practice. We then confirmed the validity and reliability of our instrument in clinical practice. This part of our study is ongoing and will be reported on in the future.

## Methods

### Phase I: literature review

We conducted a semi-systematic literature review of clinical measurements used to establish disease activity in RA patients. The literature review was conducted in Medline using the following search strategy: ((“Patient Outcome Assessment”[Mesh]) AND (“Patient Reported Outcome Measures” [Mesh])) AND (“Arthritis, Rheumatoid”[Majr]). The search was restricted to papers published in the last 5 years.

Titles of retrieved articles were screened to define whether they were suitable for our purposes, and those that were considered inadequate were excluded. This initial search was followed by similar screening processes to evaluate the abstracts in a second stage and, finally, the full text. At each of these stages, texts that did not meet the inclusion criteria were excluded. Additional articles that were relevant to the objectives of our paper were subsequently added based on the judgment and experience of the authors.

### Phase II: identification and categorization of disease activity measures

We extracted the disease activity measures reported in each article and determined the number of articles that mention each one. Next, we divided the disease activity measures into categories based on whether they required medical input, whether they might be used when a physical examination is not possible, and whether they were laboratory test values and clinical findings suggesting disease activity.

The findings were categorized into the following groups:

- Physician’s assessment of the disease
- Patient’s self-assessment of the disease (i.e., PROMs)
- Tender joint count (TJC)
- Swollen joint count (SJC)
- Laboratory tests
- Morning stiffness
- Pain
- Self-report of flare
- Full assessment scales

The full set of items found in literature as well as their frequency of appearance can be found in Table 1.

**Table 1:** Presence of clinimetric variables (percentage) in the final set of retrieved articles

Variable	Percentage
28-joint Disease Activity Score (DAS28)	26.18
C-reactive protein (CRP) – quantitative measurement	21.89
Erythrocyte sedimentation rate (ESR) – quantitative measurement	13.30
Clinical Disease Activity Index (CDAI)	10.30
Tender joint count (TJC)/swollen joint count (SJC) 28	8.58
Simplified Disease Activity Index (SDAI)	6.01
Flare self-report	5.58
Patient Global Assessment (PtGA)	5.58
Examiner-reported synovitis	4.72
Examiner-reported joint tenderness	4.29
Patient-reported pain	4.29
Evaluator Global Assessment (EGA)	3.00
Routine Assessment of Patient Index Data 3 (RAPID3)	3.00
Rheumatoid Arthritis Disease Activity Index (RADAI)	3.00
Patient-reported synovitis	2.58
Patient-reported stiffness	2.58
Rheumatoid Arthritis Impact of Disease (RAID)	2.15
Patient-reported joint tenderness	1.72
66/68-Swollen and Tender Joint Counts (SJC66/TJC68)	1.72
44-joint Disease Activity Score (DAS44)	1.72
ACR/EULAR Boolean remission criteria	1.29
Rheumatoid Arthritis Disease Activity Index 5 (RADAI 5)	1.29
Physician Global Assessment (PhGA)	1.29
Disease Activity Score (DAS)	1.29
Rheumatoid Arthritis Flare Questionnaire (RA-FQ)	0.86
Self-reported fatigue	0.86
Patient Activity Scale (PAS) II	0.86
Rapid Assessment of Disease Activity in Rheumatology (RADAR)	0.86
Visual analog scale (VAS) for disease activity	0.86
Self-reported severe inflammation	0.43
Flare instrument	0.43
Patient activity scale (PAS)	0.43
Interleukin 6 (IL-6)	0.43
Bath Ankylosing Spondylitis Activity and Function Indices (BASDAI)	0.43
Flare Rheumatoid Arthritis (FLARE-RA)	0.43
Electronic Routine Assessment of Patient Index Data 3 (eRAPID3)	0.43
Patient-based Disease Activity Score (PDAS)	0.43

*ACR* American College of Rheumatology, *EULAR* European League Against Rheumatism

### Phase III: consensus-based development of a clinimetric tool

After collecting and categorizing the variables of interest, we invited experts in rheumatology to participate in a consensus using the nominal group technique. The goal of this consensus was to determine the variables that were the most indicative of disease activity for each category according to the experts’ opinion. For patient-reported outcomes, the variables chosen were those that did not require the physician’s input.

Seventeen experts accepted the invitation to participate. Two rounds were carried out. The first round was to collect individual responses, and the second round was to share a summary of the responses provided by other participants. Finally, a vote was held in two synchronous sessions using a digital platform.

The basic questionnaire consisted of 12 sections that began with the question: “*Which item or items would you include in a clinimetric tool for use in telemedicine or data collection in the waiting room?”* followed by answer options based on the categories described in phase II. The voting process was always anonymous, and the summary of answers was subsequently sent to other experts as feedback. Once the voting process was complete, the responses were grouped by frequency according to the information provided by the experts for each group of questions.

## Results

### Literature review

**Fig. 1:**
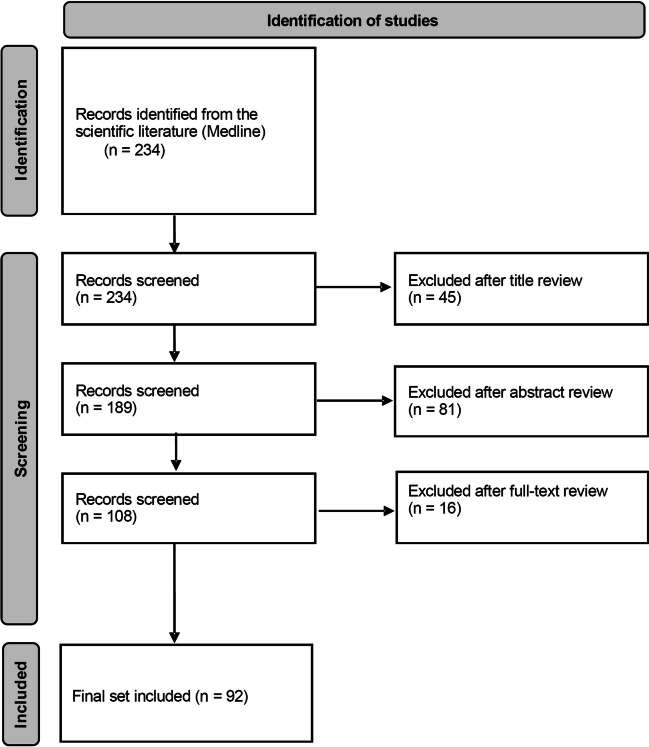
Screening process. Based on: Page MJ, McKenzie JE, Bossuyt PM, et al. The PRISMA 2020 statement: an updated guideline for reporting systematic reviews. BMJ. 2021 Mar 29;372:n71. https://doi.org/10.1136/bmj.n71

The initial literature search retrieved 234 titles. Studies that did not fulfill the search objectives were excluded. After a review of titles, abstracts, and full texts, the final set included 92 articles. Figure 1 shows a flow diagram of the records included and excluded (Fig. 1).

The reasons for excluding articles at each of the three screening stages (title, abstract, and full-text review) are shown in the Appendix.

### Disease activity measures

The most frequently reported measures of disease activity included in the final set were the DAS28, which was reported in 26.18% of articles, C-reactive protein (CRP) in 21.89%, and the erythrocyte sedimentation rate (ESR) in 13.30% (Table 1).

Within the activity indices assessed by the physician, we found 14 activity measures that required the physician’s evaluation: the most frequently reported were DAS28 (31.77%) and the CDAI (12.5%).

We identified 12 PROMs. The most common were the Patient Global Assessment (PtGA) (6.77%) and the Routine Assessment of Patient Index Data (RAPID3) (3.65%). Within this category, the most widely reported laboratory test was CRP (61.45%).

Across all the categories, the most frequently reported sign of disease was the SJC (52.38%). The most frequently self-reported symptom was increased activity or flare (26.53%).

### Consensus-based development of a clinimetric tool

**Fig. 2:**
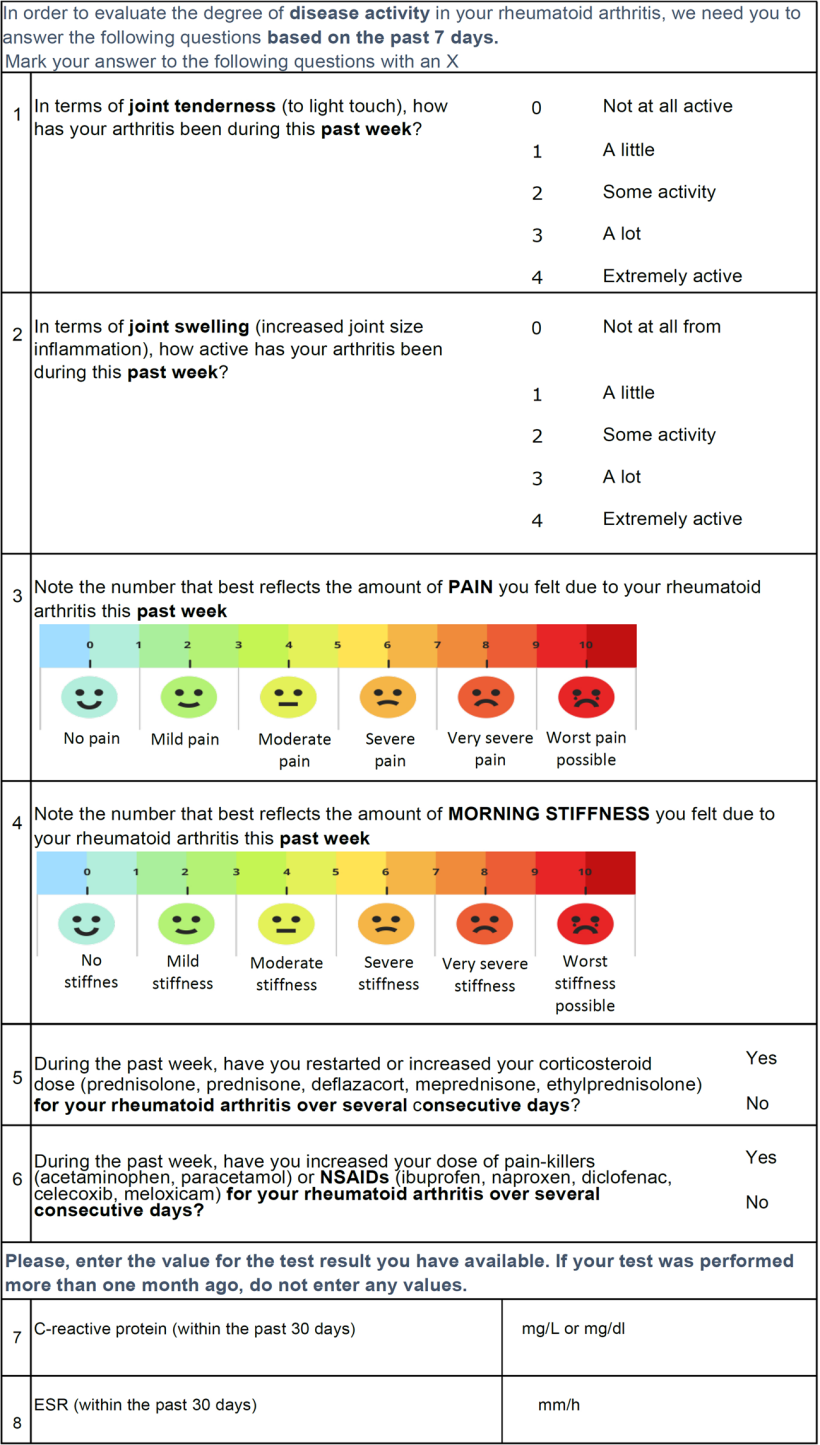
Proposed clinimetric tool for rheumatoid arthritis. PARDAI-RA by PANLAR

With the results of the consensus and the vote, we created a comprehensive clinimetric instrument that brought together the variables that ranked highest in the voting process. The resulting clinimetric tool was named **PA**tient **R**eported **D**isease **A**ctivity **I**ndex in **R**heumatoid **A**rthritis (PARDAI-RA) and can be seen in Fig. 2.

## Discussion

We collected up-to-date information on clinimetric instruments and their usefulness in RA through a semi-systematic search. This information was then used to design a comprehensive clinimetric instrument based on the most frequently used measures of disease activity (both physician- and patient-reported).

The development of a clinimetric tool for rheumatic diseases is a complex process that requires various steps to be followed [7–11, 31–33]. Several tools have been developed in recent decades (e.g., the Rheumatoid Arthritis Distress Scale of Silke et al. [32] and the Rheumatoid Arthritis Symptom and Impact Questionnaire of Becker et al. [33]), although there is still an unmet need for new measures.

The clinimetric perspective facilitates clinical decision-making. Implementation of these decisions is likely to improve outcomes both in clinical research and in practice. As new treatments and clinical concepts are constantly being developed, novel instruments and tests are needed to provide meaningful information that complements the work of the clinician. In any given medical context, including rheumatology, measurements must be accurate before tests are applied. Clinimetric properties indicating that the test is reliable and valid are essential when determining the measurement quality of any tool [6–11].

The clinimetric tools used in RA differ from those used in other clinical conditions because there is no single “gold standard” measure that can be applied to all patients. The use of multiple health domains in RA has led to the development of composite indices consisting of different quantitative measures that improve clinical evaluations by reducing measurement error, thus providing a more objective means of assessment [6–9, 9, 10, 10, 11, 11–19].

There is consensus that inflammation in RA should be controlled as soon and as completely as possible in clinical practice. Considering that the goal of treatment is to achieve the best possible control of inflammation, or even remission, the management of RA should include systematic and regular evaluation of inflammation and health status. Control of the long-term consequences, especially disability and joint damage, is a key objective in the clinical management of RA [16–20]. Our comprehensive clinimetric tool could help clinicians reach these therapeutic goals.

For the purposes of our study, we evaluated the most widely recommended composite measures of disease activity. The most common metric was the DAS28, which indicates how active RA is at the moment of the evaluation, and how it will progress over time. DAS28 has been extensively validated and is widely considered the best option for measuring disease activity in RA. It is endorsed by the American College of Rheumatology and the European League Against Rheumatism for clinical trials [13, 14]. The instrument was initially designed for comparing clinical trial outcomes of RA treatments, although these indices are now also used as overall markers of disease activity in daily clinical practice [15–19].

Response to treatment can be assessed more objectively using the TJC, SJC, or DAS evaluations. The DAS28-ESR describes the severity of RA using clinical and laboratory data and may be combined with a general health evaluation or a Patient Global Assessment (PtGA) [12–19].

We also found the CDAI and SDAI to be widely used. The CDAI combines single measures into an overall continuous measure of disease activity. It includes the 28 SJC, the 28 TJC, a PtGA based on a 10-cm visual analog scale (VAS), and the Physician Global Assessment, which is also based on a 10-cm VAS. SDAI has been extensively validated and has shown high sensitivity and specificity for predicting how physicians will modify therapy. Both indices are well-known and widely used in research and in clinical settings [10, 12–15, 19].

C-reactive protein (CRP) proved to be an appropriate alternative to ESR for assessing disease activity. Therefore, we also included CRP in our clinimetric tool. Many experts consider CRP to be a more direct measure of inflammation than ESR, with faster increases when inflammatory damage appears. Hence, CRP is considered valid as ESR for measuring the activity of RA. ESR and CRP are both associated with radiological progression in RA and are extremely useful in the monitoring of disease activity. Since the activity of RA can fluctuate between visits, close monitoring is helpful, especially when exacerbation is related to radiologic progression and structural changes.

In the development of our clinimetric tool, we gave considerable relevance to patient-reported variables. PROMs represent a significant advance in the assessment of RA. They are used as secondary outcomes in most clinical trials and are recognized as measures of treatment efficacy by the US Food and Drug Administration and the European Medicines Agency [13, 14]. Combining PROMs with objective measures provides essential insight into treatment effects and global health status [17–20].

PROMs allow patients to report their symptoms directly, although their role in assessing inflammation and joint damage may be imprecise. Symptom severity (e.g., pain and stiffness), global patient assessment, physical function, and global quality of life are essential outcomes in RA that can be measured using PROMs [20–28].

PROMs also enable the impact of treatment to be consistently quantified from the patient’s perspective and complement physician-reported measures, such as joint counts and laboratory data. Therefore, our tool included two scales to assess pain and morning stiffness from the patient’s perspective.

## Conclusion

Various indices, scales, and scores are needed for the assessment of patients living with RA, since clinical experience has shown that a single metric is unlikely to capture changes in disease activity across all RA patients in various clinical situations. Therefore, the development of a comprehensive clinimetric tool is an appropriate and essential step towards better care and management of RA patients.

In research and daily practice, disease activity is assessed using a combination of data, such as clinical variables, laboratory testing, and patient-reported variables. These categories of variables were included in our novel clinimetric tool, thus supporting its relevance in patient assessment and care.

### Supplementary Information

Below is the link to the electronic supplementary material.

Supplementary file1 (DOCX 18 KB)
