# Supplementary material for: Development of a novel clinimetric tool: PAtient Reported Disease Activity Index in Rheumatoid Arthritis (PARDAI-RA) by PANLAR, for the assessment of patients living with rheumatoid arthritis
Source: Clin Rheumatol. 2024 Feb 14;43(4):1277–85. doi: 10.1007/s10067-024-06868-w (PMC10944809; doi:10.1007/s10067-024-06868-w)
Supplement: Supplementary file 1 — Supplementary file1 (DOCX 18 KB) [file 10067_2024_6868_MOESM1_ESM.docx]

**Appendix. Reasons for exclusion of articles during the screening process.**

Forty-five articles excluded by title review:

- 2 studies compared scales between different diseases, without discussing self-reported measures of disease activity

- 1 study evaluated outcomes of discontinuation of anti-TNF agents in pregnant patients, without evaluating patient-reported outcomes

- 4 studies evaluated images in patients with RA

- 4 studies reported postoperative outcomes in patients with RA

- 3 studies discussed pain therapies in patients with RA

- 2 studies evaluated exercise as a therapeutic method in patients with RA

- 6 studies placed greater emphasis on comorbidities than on RA

- 2 studies referred to pharmacological adherence in patients with RA

- 1 study evaluated treatment disparities related to the insurance status of patients with RA

- 19 studies referred to outcomes reported by patients with diseases other than RA

- 1 study consisted of the translation of a clinimetric scale.

Eighty-one articles excluded by abstract review:

- 1 study made a comparison between clinimetric scores, without mentioning the items evaluated.

- 1 study compared outcomes according to the route of drug administration in patients with RA

- 4 studies evaluated the correlation between clinimetric scales

- 1 study evaluated maternal-fetal outcomes in patients with RA

- 3 studies evaluated postoperative outcomes in patients with RA

- 1 study evaluated pain intensity in patients with RA

- 5 studies discussed the methodological quality of clinimetric scales in patients with RA

- 1 study evaluated the effects of discontinuation of therapy in patients with RA

- 3 studies discussed the use of technology in patients with RA

- 55 articles did not mention disease activity in patients with RA

- 5 studies evaluated quality of life in patients with RA

- 1 study reported safety outcomes associated with therapy in patients with RA

Sixteen articles excluded after full-text review:

- 1 study evaluated the association between objective and subjective measures of activity evaluated by the physician

- 1 study excluded patients with signs of increased disease activity

- 1 study assessed disease activity without describing how the presence of activity was defined

- 12 studies did not analyze disease activity in patients with RA

- 1 study comprised opinions regarding clinimetric tools
